# Surgical Dressings

**Published:** 1889-12-21

**Authors:** 


### SURGERY.

### SURGICAL DRESSINGS.

Dr. Charles W. Brown, Elmira, New York, publishes an article in the last number to hand of the *New York and Philadelphia Times and Register* on "Surgical Dressings." He recommends before beginning any operation cleansing the part and hands of the operator with bichloride solution, while the instruments should be placed in a solution of carbolic acid and oil eucalyptus. Good drainage of a wound is said to be as potent to its cleanliness as good drainage of a dwelling to the health of the occupants; and for this he prefers horse-hairs or a few strands of silk-worm-gut, or catgut for the great majority of cases. The general use of drainage tubes is not considered satisfactory. For convenience in preparing a bichloride solution, the antiseptic tablets manufactured by Wyth, are said to answer the purpose very well. To produce anæsthesia the author only uses Squibb's ether. He always uses a fresh can, and he gives whisky previous to etherisation. The ether is given in the old fashion, from a folded towel. Lastly, the author gives the contents of his dressing-case, which is kept at the railroad guard office. The case contains absorbent lint, absorbent cotton, rubber, adhesive plaster, silkworm-gut, catgut, horsehair, antiseptic tablets, whisky, ether, crystals of carbolic acid, and oil eucalyptus, Esmarch's bandage, cotton bandage, rubber tubing for syphon, sponges, drainage tubes. Although there is nothing very novel in all this we have thought it desirable to "make a note" of it, as showing how our cousins manage such affairs. Whether we do not achieve the purpose quite as well at home may be open to question.

